# *Bacillus velezensis* LG37: transcriptome profiling and functional verification of GlnK and MnrA in ammonia assimilation

**DOI:** 10.1186/s12864-020-6621-1

**Published:** 2020-03-06

**Authors:** Guangxin Liu, Sarath Babu Vijayaraman, Yanjun Dong, Xinfeng Li, Binda Tembeng Andongmaa, Lijuan Zhao, Jiagang Tu, Jin He, Li Lin

**Affiliations:** 1 State Key Laboratory of Agricultural Microbiology, College of Fisheries and College of Life Sciences, Huazhong Agricultural University, Wuhan, 430070 Hubei Province China; 2 Guangzhou Key Laboratory of Aquatic Animal Diseases and Waterfowl Breeding, Guangdong Provincial Key Laboratory of Waterfowl Healthy Breeding, College of Animal Sciences and Technology, Zhongkai University of Agriculture and Engineering, Guangzhou, 510225 Guangdong China; 3 Laboratory for Marine Fisheries Science and Food Production Processes, National Laboratory for Marine Science and Technology, Qingdao, 266071 Shandong China

**Keywords:** *Bacillus velezensis*, Transcriptome, Transporter, CRISPR-Cas9, Ammonium nitrogen, Ammonia assimilation pathway

## Abstract

**Background:**

In recent years, interest in *Bacillus velezensis* has increased significantly due to its role in many industrial water bioremediation processes. In this study, we isolated and assessed the transcriptome of *Bacillus velezensis* LG37 (from an aquaculture pond) under different nitrogen sources. Since *Bacillus* species exhibit heterogeneity, it is worth investigating the molecular mechanism of LG37 through ammonia nitrogen assimilation, where nitrogen in the form of molecular ammonia is considered toxic to aquatic organisms.

**Results:**

Here, a total of 812 differentially expressed genes (DEGs) from the transcriptomic sequencing of LG37 grown in minimal medium supplemented with ammonia (treatment) or glutamine (control) were obtained, from which 56 had Fold Change ≥2. BLAST-NCBI and UniProt databases revealed 27 out of the 56 DEGs were potentially involved in NH_4_^+^ assimilation. Among them, 8 DEGs together with the two-component regulatory system GlnK/GlnL were randomly selected for validation by quantitative real-time RT-PCR, and the results showed that expression of all the 8 DEGs are consistent with the RNA-seq data. Moreover, the transcriptome and relative expression analysis were consistent with the transporter gene *amtB* and it is not involved in ammonia transport, even in the highest ammonia concentrations. Besides, CRISPR-Cas9 knockout and overexpression *glnK* mutants further evidenced the exclusion of *amtB* regulation, suggesting the involvement of alternative transporter. Additionally, in the transcriptomic data, a novel ammonium transporter *mnrA* was expressed significantly in increased ammonia concentrations. Subsequently, OE*mnrA* and Δ*mnrA* LG37 strains showed unique expression pattern of specific genes compared to that of wild-LG37 strain.

**Conclusion:**

Based on the transcriptome data, regulation of nitrogen related genes was determined in the newly isolated LG37 strain to analyse the key regulating factors during ammonia assimilation. Using genomics tools, the novel MnrA transporter of LG37 became apparent in ammonia transport instead of AmtB, which transports ammonium nitrogen in other *Bacillus* strains. Collectively, this study defines heterogeneity of *B. velezensis* LG37 through comprehensive transcriptome analysis and subsequently, by genome editing techniques, sheds light on the enigmatic mechanisms controlling the functional genes under different nitrogen sources also reveals the need for further research.

## Background

For the sustenance of life, every organism should continuously absorb various minor to major nutrients from the environment that includes carbon, nitrogen, phosphorus, sulfur and iron elements, and a wide array of other molecules to synthesize proteins, phospholipid, and nucleic acids. These molecules are required for growth and reproduction, etc., [1, 2]. In recent decades, ecosystems were increasingly contaminated by nitrogen compounds through anthropogenic exploitation, which might lead to advancing aquatic ecological issues, such as algal blooms, eutrophication, and reduced water quality [3–5]. Inorganic nitrogen usually exists in the form of molecular ammonia (NH_3_°), ionic ammonium (NH_4_^+^), nitrite (NO_2_^−^) and nitrate (NO_3_^−^) nitrogen. When, in solution, ammonia remains as NH_4_^+^ and NH_3_° forms depending on the physical and chemical factors of the aquatic environments [6–8]. The reduced nitrogen (R–NH_2_) is primarily converted into reduced NH_4_^+^ through the decomposition action of microorganisms, a process called ammonification. NH_4_^+^ is hydrated and less toxic than NH_3_°, which is fat-soluble and thus can penetrate through the cell membrane in turn affecting economic value of aquatic animals [7, 9, 10]. Accumulation of ammonia in the body could lead to “acute ammonia intoxication” by disrupting the proton gradients and the central nervous system (CNS); besides, loss of equilibrium, hyperexcitability, decreased oxygen-carrying capacity, thickening of mucous cells, liver tissue edema, and in extremis, convulsions, coma, and death [11–13]. Thus, the removal of ammonium compounds from water ecosystems is essential for animal culture applications.

In recent years, various industrial effluent treatment plants are managed with different approaches, in terms of physical, chemical, and biological methods. However, the methods comprising physical and chemical procedures require more energy and have risks of ecotoxicity, whereas the biological process is the most effective and recommended [8]. Among the various biological forms of life, bacteria serve as the conventional basis for the biological treatment of water to metabolize organic pollutants and turn them into non-toxic metabolites [14, 15]. Bacteria used in remediation processes include the following genera: *Achromobacter, Acinetobacter, Alcaligenes, Arthrobacter, Bacillus, Corneybacterium, Desulfitobacterium, Flavobacterium, Geobacterium, Micrococcus, Mycobacterium, Nocardia, Pseudomonas, Rhodococcus, Sphingomonas, Vibrio*, etc., [16–18]. In particular, *Bacillus* species demonstrate outstanding efficiency in the water restoration projects with multiple benefits, including distribution, easy isolation and cultivation, and endospores that can be stored for protracted periods [19]. However, *Bacillus* respond to N-availability by displaying heterogeneity and autoregulation through both positive and negative feedback switching mechanisms in isogenic cell populations. Regulation of genes involved in signal perception, transmembrane transporter, transcriptional regulators, and enzymatic conversion in N-metabolism [20, 21].

**Fig. 1:**
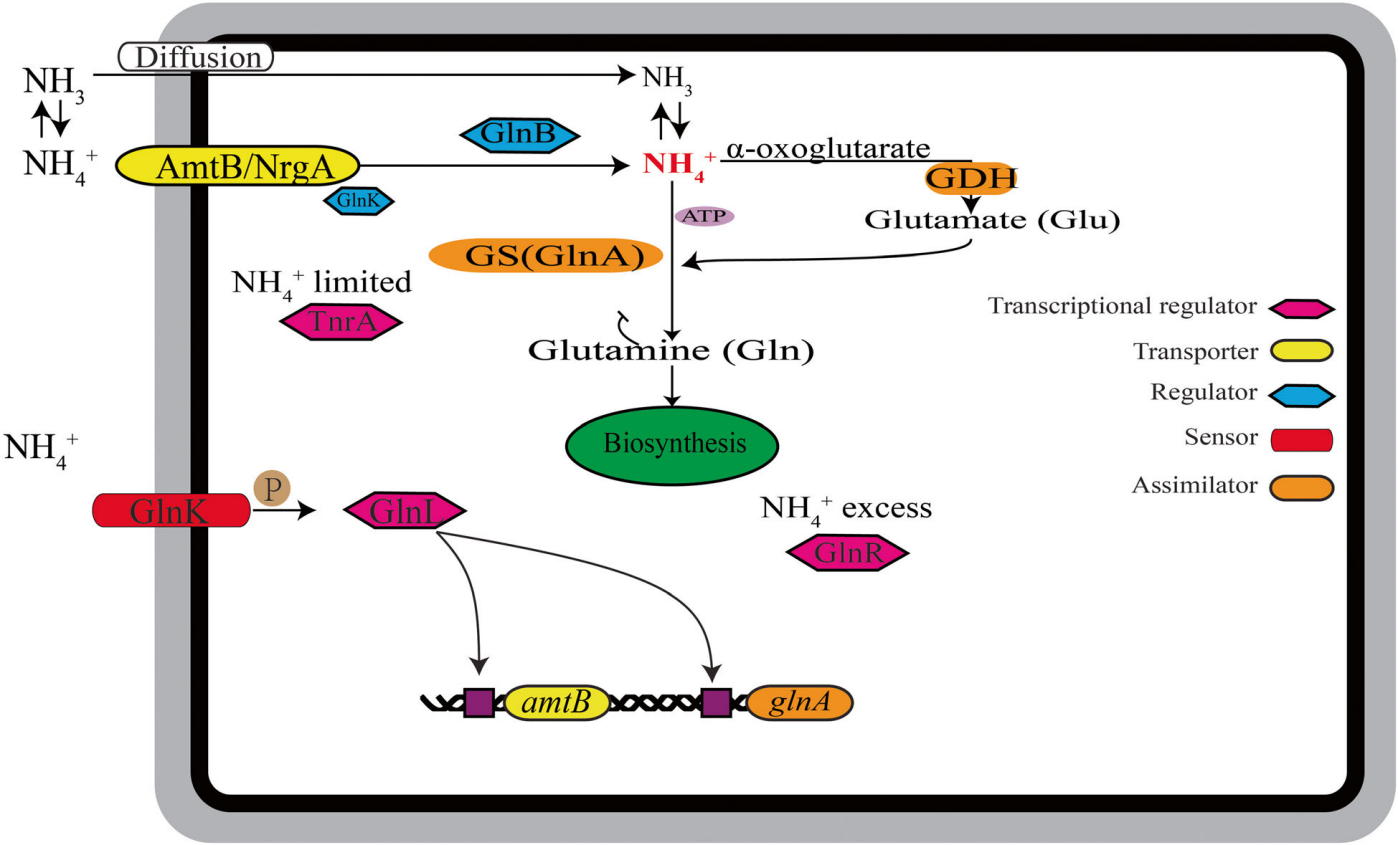
Overview of NH_4_^+^ assimilation pathway: transcriptional regulators (GlnL, GlnR and TnrA - Pink); transmembrane transporter (AmtB - yellow); assimilation regulator (GlnK and GlnB - blue); sensor (GlnK - red); assimilator (glutamine synthetase (GS)-GlnA, glutamate dehydrogenase GDH - orange)

*Bacillus* assimilates NH_3_ by diffusion under high pH and high NH_4_^+^ concentration, while intracellular transport of NH_4_^+^ occurs at low pH and low NH_4_^+^ level for assimilation under the influence of an ammonium transporter (AmtB) (Fig. 1) [22]. Thereupon, AmtB mediates and maintains ammonium homeostasis during the growth [22–24]. At high NH_4_^+^ concentrations, AmtB combines with a small cytoplasmic signal transduction nitrogen regulatory protein GlnK (sensor, histidine kinase) and forms [AmtB*-*GlnK] complexes that inactivate both the transport function of AmtB and autophosphorylation activity of GlnK [25]. Conversely, at high NH_4_^+^ concentrations, AmtB does not bind to GlnK, leading AmtB to transport the NH_4_^+^ efficiently; meanwhile, the signal transduction proteins GlnK and GlnL (transcriptional regulatory protein) form a two-component regulatory system GlnK/GlnL. The autophosphorylated GlnK transfers a phosphoryl group to GlnL, and that positively regulates the expression of the *glsA-glnT* (encoding glutaminase, glutamine transporter, respectively) in response to the intracellular concentration of glutamine for nitrogen assimilation [26–28].

The GlnA is crucial in an array of biochemical functions, including, as a transcription coregulator, nitrogen assimilator, and as a chaperone in N-assimilation based on the N-availability via regulating the expression of N-metabolism related genes [29]. Through catalysis by ATP-dependent condensation with glutamate, GlnA (glutamine synthetase) detoxifies NH_4_^+^ to Glutamine (Gln) [29–31]. As a feedback repressor, GlnA directly interacts and forms a complex with TnrA (a transcriptional regulator) in regulating the nitrogen assimilation efficiency [32–34]. TnrA acts as an activator during N-limitation and promotes expression of the genes [*amtB*, *nrgBTGH* (encoding nitrate transporter), *nasABCDEF* (encoding nitrate/nitrite assimilation), *glnA* and represses the genes *glnR* (encoding transcriptional regulator), *gltAB* (encoding glutamate synthase)] that are involved in the N-transport and metabolism [35–38]. Contrastingly, under N-excess conditions, GlnR and TnrA are active that resulting in repression of N-assimilation related genes. The N-terminal amino acid sequences in the DNA binding domains of both TnrA and GlnR are highly-homologous sequences (17 nucleotides) at the minimal binding site [37, 39, 40]. In the genus *Bacillus*, the regulatory mechanisms for N-metabolism are very diverse in response to the different concentration and the different forms of N-sources at altered conditions through specific strategies, to realize the assimilation of N-sources by cells [22].

Considering all the above, in this study, a new *Bacillus* sp*.,* efficient in N-assimilation, was isolated from an aquaculture pond. The genomic sequence profiling (Genbank Accession Number: CP023341.1) revealed it is a *Bacillus velezensis* strain and named as *Bacillus velezensis* LG37 (hereafter as LG37). The fascinating species *B. velezensis* was already acknowledged its potential in exerting the ammonia metabolism [41].

We analyzed the growth characteristics and excavated the metabolic pathway-related genes by transcriptomic profiles of LG37 cultivated under different sole N-sources [inorganic nitrogen (NH_4_^+^) and organic nitrogen (glutamine)] using high-throughput Illumina sequencing technology. Moreover, following the transcriptomic data, the CRISPR-Cas9 genome editing system has been applied to the related genes to validate their specific regulation mechanism effectively. The results showed that GlnK plays an essential role in ammonia metabolism, and AmtB as a transporter does not represent an influential role in ammonia transport. Meanwhile, we found a new ammonia transporter (MnrA) in *B. velezensis* LG37 and verified its function underlying the pattern of ammonia transport. The obtained transcriptomic data and molecular editing of specific genes by CRISPR/Cas9 in *B. velezensis LG37* for ammonia metabolism will shed new light on the microbial ammonia assimilation.

## Results

In the present study, we isolated three *Bacillus* strains from a grass carp pond water. Among them, a particular isolate, namely, *Bacillus velezensis* LG37, was successfully screened using minimal media with ammonia as the sole nitrogen source for the best ammonia nitrogen assimilation efficiency; thus, it was chosen for further analysis.

### **Growth characteristics of** LG37 culture conditions

The bacterial growth kinetics of wild-type LG37 was analyzed by culturing in LB broth, and the growth was measured by spectrophotometry. From 25 to 37 °C, the LG37 showed an excellent growth trend. The peak levels OD (≈7) were maintained for almost 30 h in the static cultivation environment (Additional File 1). As shown in Additional File 1, LG37 on 1–6% of salt concentrations had a sharp and stable increase in their growth exceeding OD_600_ value of 5 at 28 h followed by a gradual decline. Yet, the levels were maintained above 5 up at to 3% salinity, and the decrease in OD was proportional to salt concentration in the extended period. The LG37 growth was normal at pH 6–8.5, while slow growth was recorded at pH 9.5. The dissolved oxygen (DO) concentration available in the medium mainly influences the growth of the bacterial population [42]. The increasing growth values were observed in all the DO reactors from 0 to 40 h, and the LG37 growth was proportional to the level of DO. Peaked OD of 8.2, 6.1, and 4 – were obtained for LG37 with initial DO of 6.0, 4.2, and 3.0 mg/L, respectively, at 35 h of the culture period. Conversely, there was no substantial growth recorded in the 1.8 mg/L DO (Additional File 1).

### Optimization of glutamine and ammonia nitrogen concentrations for LG37

**Fig. 2:**
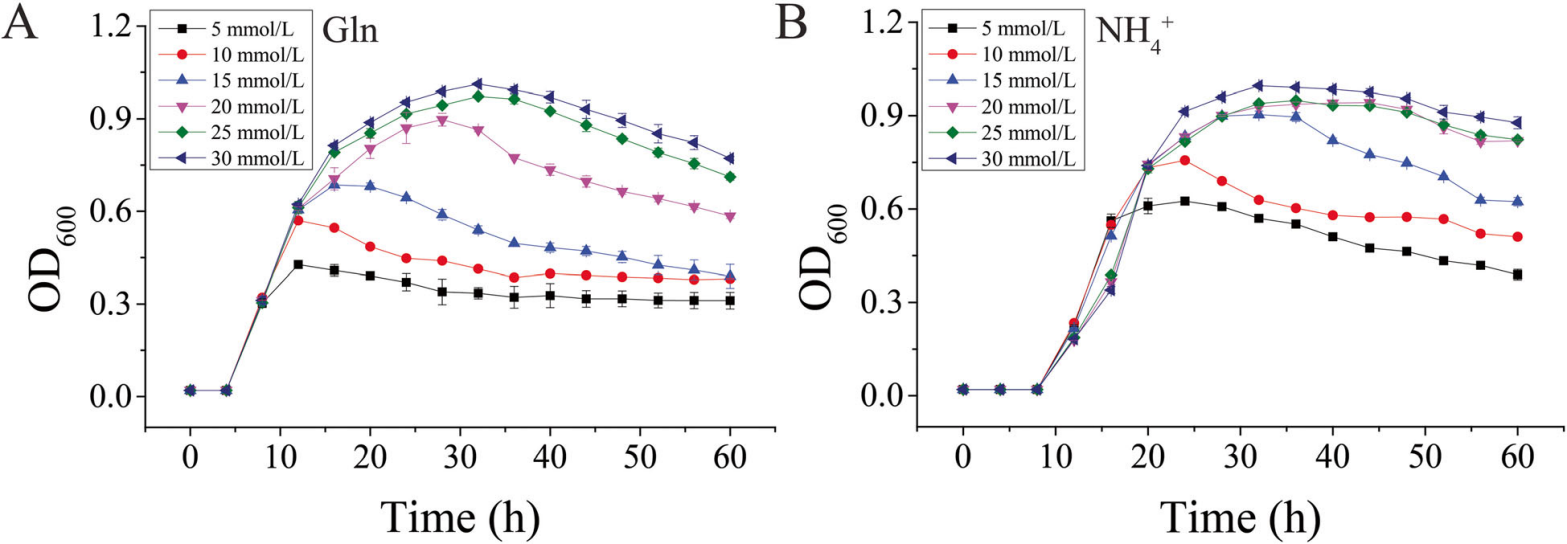
Growth curves of LG37 with different Nitrogen sources. Minimal media with various concentration of (**a**) Glutamine [Gln] and (**b**) Ammonia [NH_4_^+^] (5, 10, 15, 20, 25 and 30 mmol/L) cultured over 60 h and the OD_600_ was determined for every 4 h; all data points mean ± SE (*n* = 3)

Growth characteristics of LG37 were determined at various levels of NH_4_^+^ and Gln by OD_600_. The results showed, with the increase of Gln concentration, LG37 displayed an inclination of growth respect to the amount of Gln (Fig. 2a). The growth curve of LG37 in minimal media with NH_4_^+^ (5, 10, 15, 20, 25, and 30 mmol/L) as the sole nitrogen source showed a similar trend to that of Gln, but no significance was observed at 20–30 mmol/L NH_4_^+^ concentrations (Fig. 2b). However, the growth of LG37 was more significant in the increasing levels of nitrogen; the other nutrients in the minimal media might be a limiting factor for their growth above 20 mmol/L. Given that and to avoid overloading, we preferred an average volume (10 mmol/L) of N-source for both Gln-N and NH_4_^+^-N in our supplementary studies.

### Transcriptome assembly and functional annotation

The mRNA of the LG37 cells acquired from the treatment group (NH_4_^+^-N) and control group (Gln-N), were sequenced by Illumina Hiseq™ 2500 to obtain the overview of the gene expression pattern. After removing low quality (Q > 20) ambiguous reads from the raw data, a total of 19,091,060, 19,270,408, and 19,079,520 clean reads from the LG37-Gln (Gln-N1, Gln-N2, Gln-N3), and 18,922,170, 18,818,298, and 18,865,012 clean reads from LG37-NH_4_^+^ (NH_4_^+^-N1, NH_4_^+^-N2, NH_4_^+^-N3), were obtained, respectively. The details of GC contents, valid ratio, raw reads, and clean reads were summarized in Additional File 2. We aligned our LG37 data with the known reference genome (*B. velezensis* FZB42; Accession No: NC_009725.1) using the BWT algorithm to interpret the clean read sequences. A total of 2569 genes from LG37 were annotated as protein-coding genes; additionally, we also predicted 63 new genes, including 30 sense transcripts and 33 antisense transcripts, 2131 for 5’UTR, 2037 for 3’UTR, and 759 predicted multi-gene operons. Summary of alignment with protein-coding genes, predicted transcripts, and predicted RNAs (antisense) were presented in Additional File 3.

The LG37 DEGs potentially intricated in nitrogen assimilation were determined by conducting the statistical analysis through expression > 1.5-fold change and significant difference of *q-*value < 0.05 as standards. Expression variances were compared between the treatment and control groups using the standardization of RPKM and UQ values. In total, 812 DEGs (76 upregulated genes and 736 down-regulated genes) were screened. Among them, 56 genes met the differential expression genes (DEGs) criteria (i.e. expression value > = 2-fold and *p*-value <= 0.05). Subsequently, the genes were compared with NCBI and UniProt database for screening the candidate related genes for N-metabolism, resulted in 27 candidate genes, including 18 upregulated DEGs, and 9 downregulated DEGs, listed in Additional File 4.

**Fig. 3:**
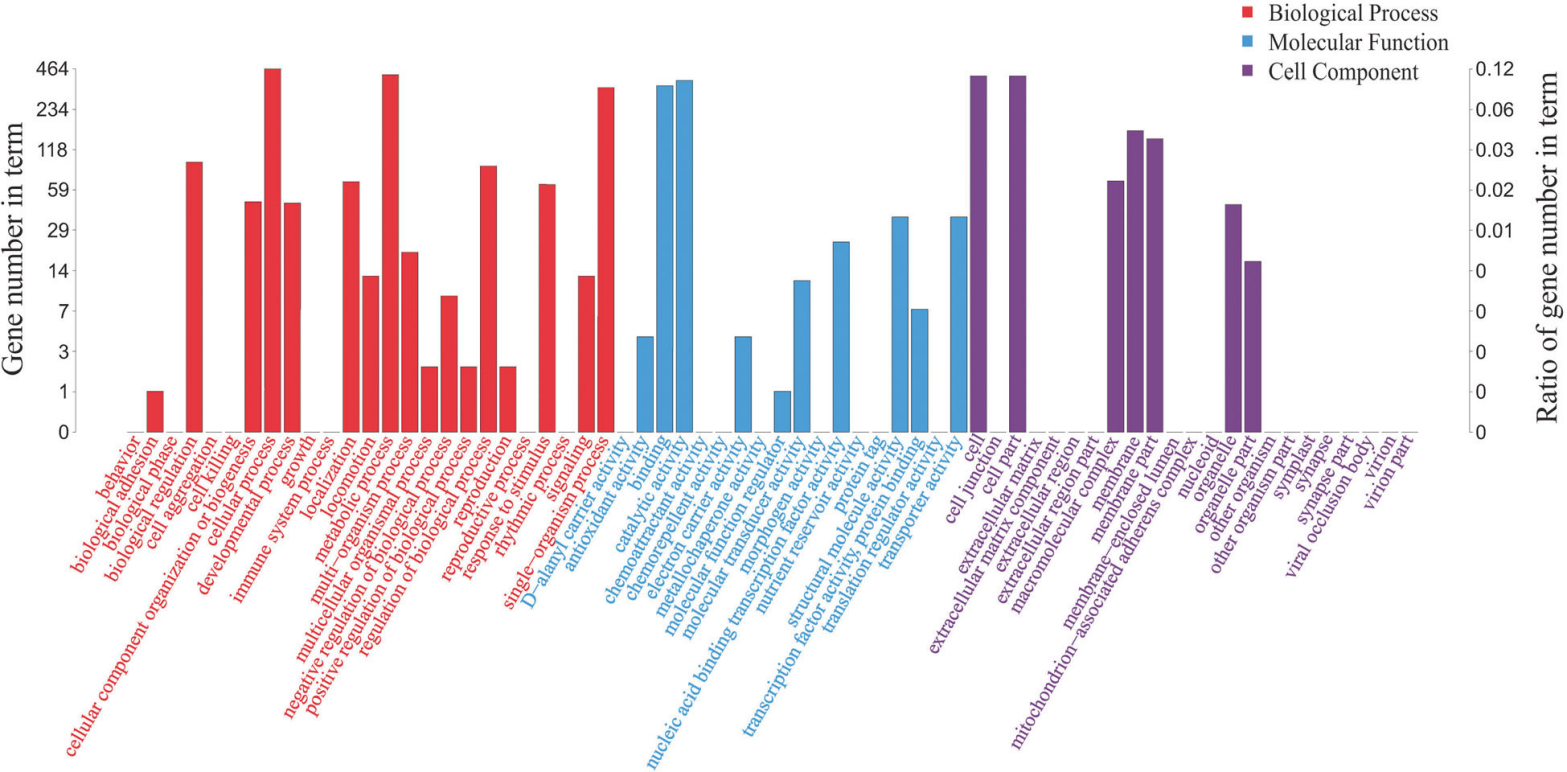
The Gene-Ontology terms and pathway enrichment analysis of LG37 differentially expressed gene (DEGs) under the cutoff of *P*-value < 0.05. The histogram showing the gene annotations that was classified into Biological process, Molecular function, and Cellular component. The vertical axis indicates the count of genes

**Fig. 4:**
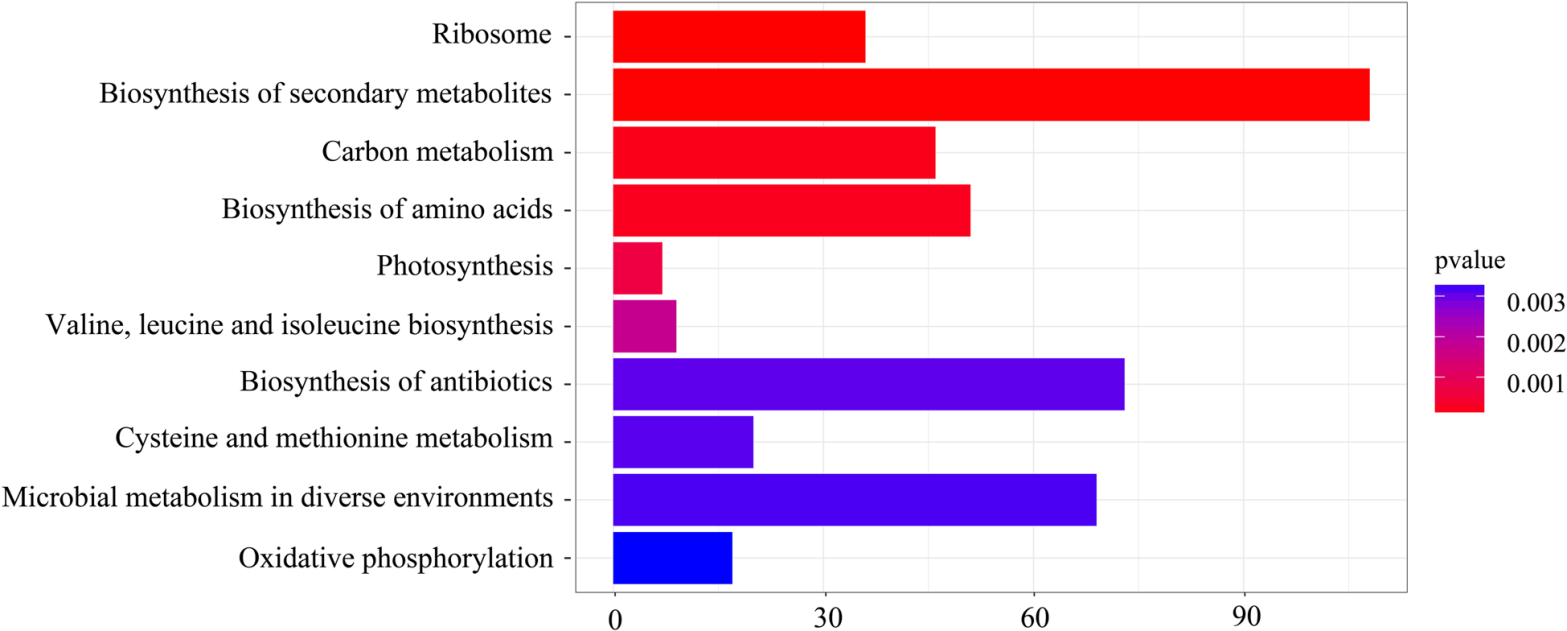
The KEGG terms and pathway enrichment results of LG37 differentially expressed gene (DEGs) with various concentrations of ammonia. The horizontal axis of the histogram indicates the count of genes and the colors of column of y-axis represents the different *P-*values of gene functional classification

The regulatory function, the expression pattern in various cellular compartments, molecular function, and biological processes of these genes in treatment and control nitrogen groups of LG37 were overviewed by mapping unto GO and KEGG databases. The results demonstrated that 812 unique proteins assigned to 3817 GO terms; 1698 unigenes mapped to biological processes, 860 unigenes mapped to molecular functions, and 1259 unigenes mapped to cellular components (Additional File 5). In the biological process subclass, the top 3 categories were “cellular process (464), metabolic process (420), and single-organism process (340)”. In molecular function, the top 2 subclasses were “catalytic activity (383) and binding (351)”, accordingly, in the cell compartment, the most abundant categories were “cell (411), cell part (411), membrane (163), and membrane part (142)” (Fig. 3). Moreover, the KEGG pathway analysis indicated the DEGs were significantly (*p*-value 0.001) enriched in five KEGG pathways, comprising pathways associated with Biosynthesis of secondary metabolites (108), Ribosome (36), Biosynthesis of amino acids (51), Carbon metabolism (46), and Photosynthesis (7) (Fig. 4, Additional File 6).

### RT-qPCR verification of selected genes

**Fig. 5:**
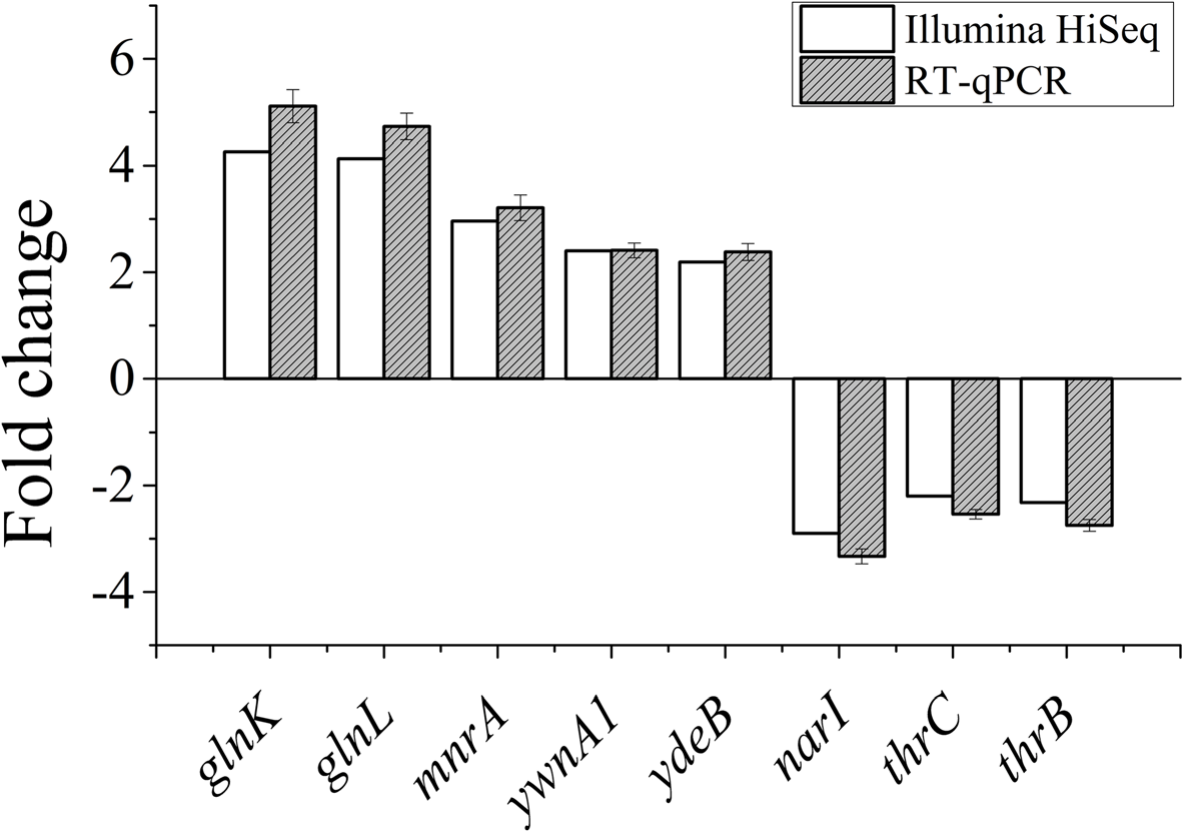
Comparison of the gene expressions of 8 DEGs cultured with NH_4_^+^ (treatment) and Gln (control) as the sole nitrogen source determined by Illumina HiSeq™ 2500 sequencing and RT-qPCR using 16S rRNA gene as reference control. The x-axis displays 8 genes and y-axis is the expression levels (fold changes). Sensor histidine kinase (*glnK*), DNA-binding response regulator (*glnL*), MFS transporter (*mnrA*), Rrf2 family transcriptional regulator (*ywnA*), CarD family transcriptional regulator (*ydeB*), Nitrate reductase subunit gamma (*narI*), Threonine synthase (*thrC*), Homoserine kinase (*thrB*). The positive and minus value means up-regulated and down-regulated, respectively to three parallel experiments

The Illumina sequence of LG37 expression profile data was verified through randomly selected 8 DEGs that including, 5 up-regulated and 3 down-regulated genes (Additional File 7) using RT-qPCR. The results exhibited a similar expression tendency but with slight variation in their levels, which confirmed the reliability of DEGs from the transcriptome sequencing results (Fig. 5).

### GlnK is critical for NH_4_^+^ assimilation

**Fig. 6:**
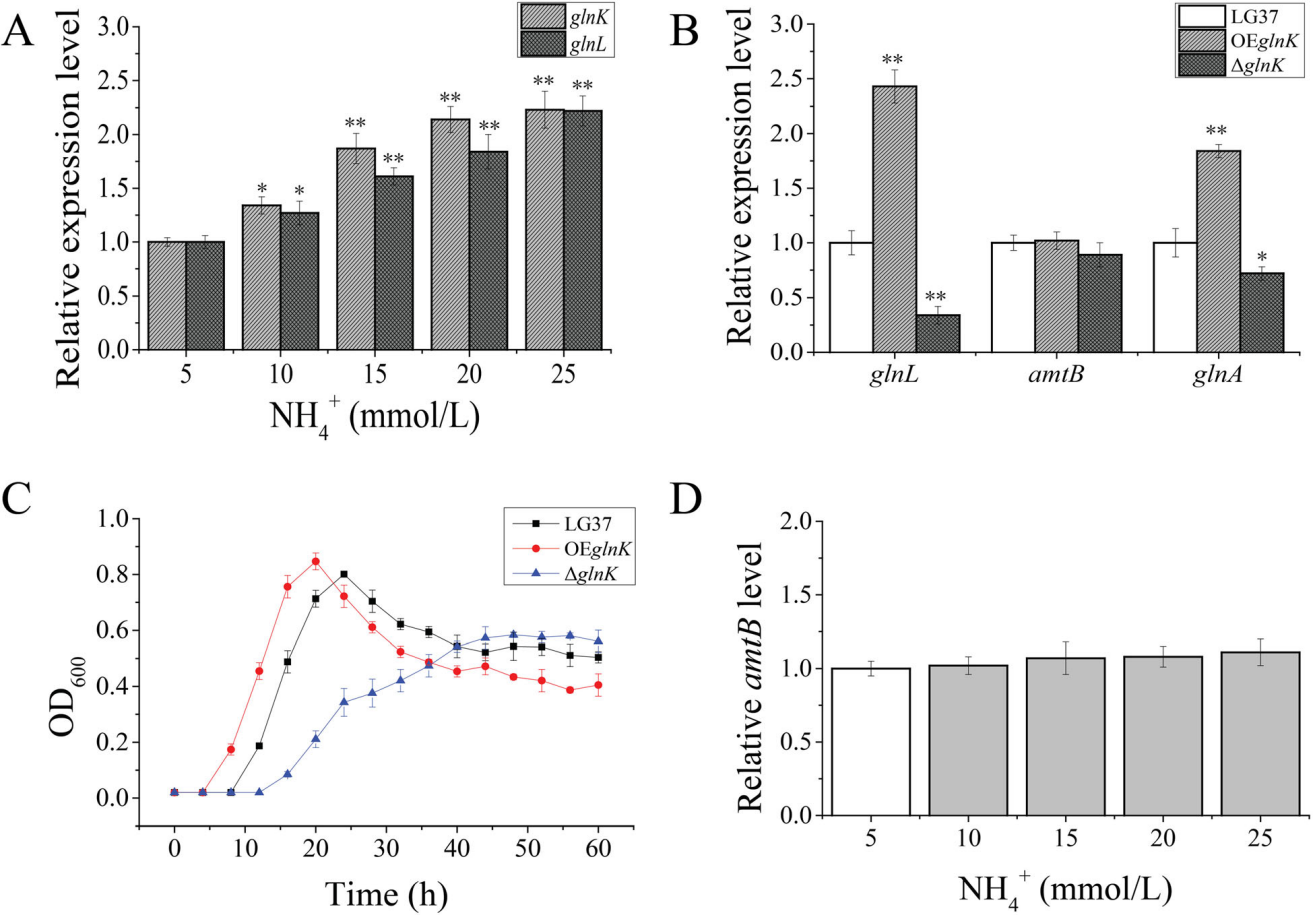
Function authentication of LG37 with *glnK* deletion (Δ*glnK*) and overexpression (OE*glnK*) by RT-qPCR analyses. **a** Relative-expression analysis for *glnK* and *glnL* of wild-LG37 cultured with different NH_4_^+^ concentration, 5 mmol/L as control. **b** Quantitative relative-expression analysis of *glnL*, *amtB,* and *glnA* in wild-LG37 and mutant OE*glnK* and Δ*glnK* strains. **c** The growth curve analysis of the wild-type and both mutant strains (Δ*mnrA* and OE*mnrA*) cultured in minimum medium with 10 mmol/L NH_4_^+^. **d** Quantitative analysis of *amtB* relative expression level of wild-LG37 cultured with increasing NH_4_^+^ concentration, 5 mmol/L as the control. These results are means ± SD. *P < 0.05, **P < 0.01 versus control

To understand the functions of the specific genes associated with NH_4_^+^ assimilation pathway that short-listed by transcriptome data, especially the significantly upregulated *glnK* (4.26) and *glnL* (4.12), were primarily analyzed by RT-qPCR following cultivation of wild-LG37 at different concentrations of NH_4_^+^. The *glnK* and *glnL* showed a significant increase in their expression with the rise of NH_4_^+^ concentration, and the *glnK* showed a greater tendency than the *glnL* (Fig. 6a). These results demonstrate that the two-component regulatory systems *glnK/glnL* play a synergistic regulation function in the LG37 NH_4_^+^ metabolism.

### Functional analysis of related genes using OE*glnK* and Δ*glnK* in NH_4_^+^ metabolism

To demonstrate the functionality of the GlnK in NH_4_^+^ assimilation pathway, *glnK* was knocked out (LG37-Δ*glnK*) by applying the CRISPR/cas9 technology (Additional File 8) using pHT1K plasmid that developed overexpression mutant (LG37-OE*glnK*) strain (Additional File 9). The mutants were analyzed for the regulation of ammonia assimilation related genes (*glnL*, *amtB,* and *glnA*) by RT-qPCR assays along with wild LG37 strain, following cultivation in 10 mmol/L NH_4_^+^ containing minimal medium. The LG37-OE*glnK* strain increased the expression of about 2.5 folds and 1.8 folds, while the LG37-Δ*glnK* strain lead to a decrease in the expression to 0.25 and 0.7 folds for *glnL* and *glnA*, respectively. No notable changes were recorded in the *amtB* gene expression compared to that of control (Fig. 6b). The growth curve of both the mutated strains represented variations in growth with increased growth in LG37-OE*glnK* and decreased growth for LG37-Δ*glnK* strain when compared to the wild-LG37 strain (Fig. 6c). These results indicated that the GlnK plays a significant role in the NH_4_^+^ assimilation of LG37.

Based on the above results, we noted that the GlnK senses the NH_4_^+^ concentration and regulates *glnL* expression through signal transduction, and further, the GlnL promotes *glnA* and *amtB* in favor of NH_4_^+^assimilation. Besides, AmtB as an ammonium transporter, there was no significant expression among the 3 groups (Fig. 6b), which is similar to that of the transcriptome results of *amtB* between NH_4_^+^-N and Gln-N nitrogen groups. Hence, we analyzed the relative expression of AmtB in wild-LG37 at an increased NH_4_^+^ concentration, and the results exhibited no difference in their expression pattern even at the highest NH_4_^+^-N levels (Fig. 6d). The above data suggest that the GlnK plays a vital role in regulating GlnL and GlnA, and still, the AmtB were not as a specific positive factor for NH_4_^+^ transporter of LG37 in assimilation. Therefore, it has been ruled out that there might be other NH_4_^+^ transporters in the case of LG37.

### Determination of the NH_4_^+^ transporter MnrA in LG37

**Fig. 7:**
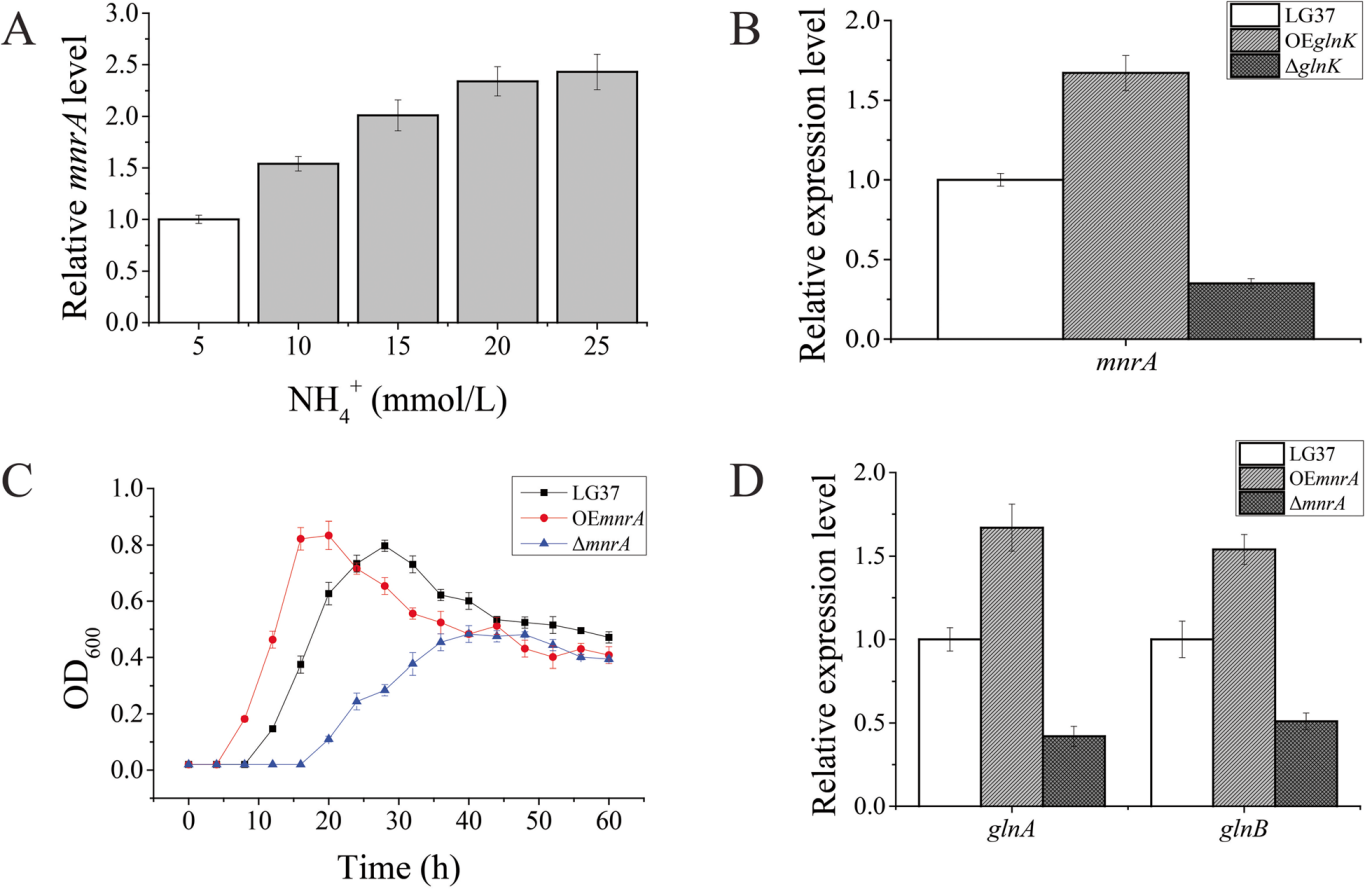
Function authentication of LG37 with *mnrA* deletion (Δ*mnrA*) and overexpression (OE*mnrA*) by RT-qPCR analyses. **a** Relative-expression analysis of *mnrA* gene in wild-LG37 with increasing concentration of NH_4_^+^ in minimal medium, 5 mmol/L as control. **b** The OE*mnrA* expression was increased and the Δ*mnrA* was hindered compared to that of wild-LG37 when cultured in minimal medium with 10 mmol/L NH_4_^+^. **c** The growth curve analysis of wild-LG37 and both mutant strains (Δ*mnrA* and OE*mnrA*) cultured in minimum medium with 10 mmol/L NH_4_^+^. **d** The downstream functional genes *glnA* and *glnB* expression was increased in OE*mnrA* and decreased in Δ*mnrA* which cultured with 10 mmol/L NH_4_^+^. These results are means ± SD. *P < 0.05, **P < 0.01 versus control

Considering the relative expression of *amtB* indicated in Fig. 6b and d, we discovered that the AmtB is not specific in the NH_4_^+^ transport of LG37, but this finding was only based on our transcriptomic and mRNA expression data and not direct assessment. Thus, we further analyzed our transcriptome data for another transporter, where we noticed mnrA with a 2.93-fold change in expression. (Additional File 4). To verify whether MnrA plays a role in NH_4_^+^ assimilation, we detected the expression of *mnrA* of LG37 after culturing with different NH_4_^+^-N concentrations by using RT-qPCR. The expression of *mnrA* increased with an increase in NH_4_^+^-N concentrations (Fig. 7a). In the LG37-Δ*glnK* and LG37-OE*mnrA* mutant strains, the *mnrA* expression was increased by a factor of 1.67 and decreased to 0.35, respectively, compared to that of wild-LG37 strain (Fig. 7b). Similarly, we determined the growth of both strains showed increased growth pattern in LG37-OE*mnrA,* whereas LG37-Δ*mnrA* strain showed a decreased growth compared to that of wild-LG37 strain (Fig. 7c). These results demonstrated that MnrA is essential for the NH_4_^+^ assimilation process in LG37.

Further, to extend our studies on MnrA, we analyzed the expression of downstream functional genes *glnA* and *glnB* in the NH_4_^+^ assimilation pathway for both LG37-OE*mnrA* and LG37-Δ*mnrA* strains. The relative-expression levels of *glnA* (1.7 folds) and *glnB* (1.6 folds) were increased in the OE*mnrA* strains, whereas, in the Δ*mnrA* strain the expression of *glnA* and *glnB* were only 0.3 and 0.5 folds, respectively (Fig. 7d). These findings demonstrated that the MnrA was served as the transporter and play a significant role in the process of NH_4_^+^ assimilation in LG37.

## Discussion

Present-day intensive aquaculture practices are often accompanied by a large volume of excretes and other organic residue accumulations, resulting in the water quality deteriorations. In particular, the building of nitrogenous compounds, namely nitrite and ammonia, are harmful to the aquatic animals, although in their least concentrations [43, 44]*.* Proper aquatic animal health management exercises begin with the maintenance of water quality. It can be achieved by assisting with various biological forms of lives that including various of *Bacillus* species. Indeed, demonstration of genetic heterogeneity nature of various *Bacillus,* not only in genetically distinct in subgroups but also within a clonal and synchronized bacterial population [6]. To date, a certain amount of *Bacillus* species such as *B. methylotrophicus* strain L7 [6], *B. velezensis-*M2 [14], *B. azotoformans* LMG 9581 T [22], *B. subtilis*, strain A1 [45], and *B. subtilis* [46] strains demonstrated substantial ammonia nitrate removal. All of those previous findings suggesting an encouraging sign, lead us to isolate the potentially efficient Bacillus strain for NH_4_^+^ assimilation and to determine the mechanism of a complex metabolic network like signal inductors, transcriptional regulators, transporters, assimilation enzyme and so on. The increased heterogenetic gene-expression of the bacterial population could not only utilize the existing nutrients for their growth but also get a benefit for the survival of the bacterial population in extreme conditions. One needs to study the interacting molecules and their networks, contribute to understanding the biological system function [47].

In the present study, a *Bacillus velezensis* LG37 was isolated from the aquaculture pond that exhibited a greater nitrogenous compound removal between the three isolated strains. The LG37 strain illustrated good growth at a wide range of temperature, pH, and salinity. For the transcriptomic approach of nitrogenous substances, we determined the growth conditions using the minimal inorganic medium that lack rich nutrients incorporated with organic (Glutamine-N) and inorganic nitrogen (NH_4_^+^-N) for ammonia nitrogen metabolism at the molecular level in LG37. Undoubtedly, the LG37 could able to grow well in both N-substrates but to bypass overstressing and to offer other nutrients from the minimal media; the substrates were volume-averaged to 10 mmol/L. Mining the potential genes from the transcriptome data may shed light on the mechanism of N-metabolism of LG37*.*

To dissect the role of differential gene expression (DEGs) profiles, a global transcriptomic profiling method was employed for the LG37 genome that cultivated under two different N-sources. Annotation for the Ammonia N-metabolism of LG37 was established using the NCBI and UniProt database and compared to that of COG and KEGG pathway following the *B. velezensis* FZB42 genome as a reference. Here, we obtained 812 DEGs (76 upregulated gene and 736 down-regulated genes) from the transcriptomic data analysis and found out 18 upregulated candidate genes, which might have involved in the NH_4_^+^ assimilation pathway. The upregulated DEGs include *glnK*/g*lnL* (two-component regulatory system), three transcriptional regulators, one for transporter and ABC transporter permease, eight unannotated functional genes and others. Many previous studies reported that the member of the two-component GlnK/GlnL (histidine kinase/DNA-binding response) regulatory system upon activation regulate the downstream genes such as the transporter *amtB* (*nrgA*) and convert the ammonia into glutamine through ATP-dependent condensation by glutamine synthetase (GS, encoded as *glnA*) [48, 49]. GlnK, as a signal-transduction protein molecule of the two-component regulatory system, senses the existence of extracellular nitrogen and activates the GlnL response regulator by the phosphoryl group [36]. Our results were in accordance with the previous studies, demonstrated that the two-component regulatory system *glnK* and *glnL* were significantly upregulated that are classified for sensor kinases (4.26 folds) and a transcriptional regulator (4.12 folds), respectively. However, the 16 DEGs that have not been reported to involve in either of the following NH_4_^+^ assimilation pathways, glutamine synthetase (GS), glutamate synthase (glutamine 2-oxoglutarate amidotransferase (GOGAT), and glutamate dehydrogenase (GDH)) NH_4_^+^ assimilation pathways which are normally used by bacteria for NH_4_^+^ assimilation. Presently, it cannot be ruled out the possibility, and further studies will address the features of the unreported functional genes involved during ammonia assimilation.

Considering the greater challenges related to the data derived from the transcriptomic study that have to be further verified by other approaches, including RT-qPCR [50, 51]. In our study, to eliminate the effects of possible amplification bias, we validated the expression patterns of the 8 randomly selected genes using the RT-qPCR. The primary results displayed that the pattern distribution and gene-expression levels were highly correlated with Illumina sequencing data. Additionally, these comparative data presented that not all the functional genes contributed in the same manner but also proved the significance of the genes in the ammonia assimilation process. Although the preliminary results provide a theoretical basis, such information still required an in-depth analysis of potential functional genes in the NH_4_^+^ assimilation metabolic network of LG37.

Following our transcriptome data, it was shown that there is no positive correlation between *glnK* and *amtB*. We speculate that the AmtB is not specific for NH_4_^+^ transport, as well as there was not a direct interaction also between GlnK and AmtB. To test the role of LG37 specific genes in NH_4_^+^ assimilation, mutant strains were constructed using the all-in-one CRISPR-Cas9 genome editing system to develop knockout (pJOE8999) [52] and overexpression (pHT1K) LG37 strains. It was again exposed that GlnK does not have a correlated mechanism with the transporter AmtB between the wild-LG37 and mutant (OE*glnK* and Δ*glnK*) strains. Whereas, the *glnL* and *glnA* expressions were significantly upregulated in the wild strain as well as in the LG37 mutants (OE*glnK* and Δ*glnK*). Similarly, growth curves of the wild-type and mutant strains demonstrated a consistent decrease in the growth of Δ*glnK* mutant; however, little difference was observed between OE*glnK* and the wild-type strain (Fig. 6c). The reduced growth of the knockout cells might be due to the inability to utilize the N-compounds where the minimal media contained only necessary essential nutrients. These results indicated that although *glnL* and *glnA* was induced, the *amtB* gene was found to be non-essential for the transport of NH_4_^+^ in LG37 strain. Thus, LG37 might likely adopted distinct NH_4_^+^ transporter mechanism similar to that of AmtB in other *Bacillus* strains.

Conversely, based on the LG37 transcriptomic data, we found a distinct transporter (*mnrA*) was upregulated to 2.92 folds, and we speculate that MnrA might be an alternate NH_4_^+^ transporter in LG37. The MnrA is a single-polypeptide secondary carrier transmembrane transporter protein belong to the Major Facilitator Superfamily (MFS) that promotes small solutes in response to chemiosmotic ion gradients [53, 54]. The relative expression levels of *mnrA* showed remarkable expression levels when LG37 grown in the increasing NH_4_^+^ concentrations, thus the same reflected in the OE*glnK* mutant strain. The Δ*glnK* mutant resulted in a reduction of *mnrA* expression compared to that of wild type.

Furthermore, to confirm this theoretical assumption, we constructed the Δ*mnrA* and OE*mnrA* LG37 strains, as mentioned earlier, and analysed for their growth and the expression of downstream genes (*glnA* and *glnB*) by RT-qPCR. Our analysis revealed that growth characteristics of the LG37 *mnrA* mutant strains were almost similar to the growth of *glnK* mutants and the contrasting expressions of *glnA* and *glnB* genes between the strains, compared to that of wild-LG37.

## Conclusions

The newly isolated bacterium *Bacillus velezensis* was named as *B. velezensis* LG37, showed its ability of well growing in various basic parameters and its heterotrophic capacity to utilize both NH_4_^+^-N and Glutamine-N as a sole nitrogen source. The transcriptome sequence analysis under different nitrogenous sources proposed that LG37 up-regulated (18) or down-regulated (9) genes predominantly related to nitrogen metabolism. Moreover, 18 up-regulated DEGs associated with a few hypothetical proteins, transcriptional regulator, transporter, transporter permease, and GlnK/GlnL regulatory system for assimilating the N-compounds. Further, we applied genome editing technology to the genes whose expression was impacted in nitrogen assimilation; the *glnK* mutants led to uncovering that *amtB* was not associated with ammonium transport (Fig. 6). However, knock out of *glnK* in LG37 showed comparable growth to wild-type/OE*glnK* on ammonia as a sole nitrogen source, which demanded us to expose the unidentified ammonia transport system. In particular, the upregulation of *mnrA* transcriptional expression was shown to correlate with the ammonia transporter specifically and exhibited the regulation of related downstream genes (*glnA* and *glnB*). Altogether, the MnrA was proved as a novel ammonium transporter in *Bacillus velezensis* LG37 and our results provide a theoretical basis and new clues to the NH_4_^+^ assimilation mechanism. Furthermore, we hypothesize that more than one ammonia transporter might have involved from the transcriptome of LG37 differential expression (Additional File 4), and this can be evaluated through subsequent experiments.

## Methods

### Bacterial isolation

The *Bacillus* sp. was isolated among a collection of microbes from the aquaculture pond in Huazhong Agricultural University teaching practice base, Wuhan, Hubei Province, China, where grass carp was cultivated, following the following operating protocol. Briefly, a series of 10-fold diluted water samples in distilled water were inoculated (100 μL) on to the LB agar plates using a sterile triangle glass rod and incubated overnight at 32 °C. Three Bacillus isolates were obtained from the pond water and characterized for morphological, physiological, and biochemical characters extensively based on the Bergey’s Manual of Determinative Bacteriology [55]. Among them, one *Bacillus* (wild) strain was identified with the highest nitrogen removal efficiency and named as *Bacillus velezensis* LG37.

### Determination of optimal nitrogen concentration and other growth characteristics

To evaluate the growth of various nitrogen substrates and optimization of culture conditions for LG37 strains. The inoculum obtained from the late logarithmic phase was inoculated in 250 mL with 100 ml of Luria-Bertani (LB) broth (Additional File 10) [56] as batches with initial optical density (OD_600_) of 0.02. The batch operations were conducted and maintained at different temperatures (25, 28, 30, 32, 35, 37 °C), pH (5, 6, 7, 7.5, 8, 8.5, 9, 9.5), salinity (1, 2, 3, 4, 5, 6%), and dissolved oxygen (DO; 1.8, 3, 4.2, 6.0 mg/L) levels in minimal medium at 32 °C, under orbital shaking at 200 rpm. For N-optimization for the growth in minimal medium, 100 ml of minimal medium containing either Ammonium Sulfate-N [(NH_4_)_2_SO_4_] or glutamine-N individually as the sole nitrogen source were inoculated at different concentrations (5, 10, 15, 20, 25 and 30 mmol/L), and incubated as mentioned above. The biomass accumulation was determined for every 4 h by measuring the optical density (OD) at 600 nm by spectrophotometer (MAPADA V-1100D, China). All tests were repeated at least three times to confirm the growth patterns.

### RNA extraction, library construction, and high-throughput sequencing

*B. velezensis* LG37 grown in a minimal medium containing 10 mmol/L NH_4_^+^-N (treatment) and 10 mmol/L Gln-N (control) and adjusted their pH to 7.0 using HCl / NaOH before inoculating the culture. At the late log phase (0.4~0.7 OD), all the samples were harvested (each sample consists of three biological repeats) from each sample. Upon harvesting, the cells were immediately frozen by liquid nitrogen without adding any the killing buffer for further transcriptome analysis. Because *Bacillus* as a gram-positive bacterium that holds a thick peptidoglycan layer which resists such reagents for a while before acting upon, and the intended period or the mixtures might affect the nature of gene expression. The frozen bacteria were pulverized carefully in a mortar using a pestle with a constant supply of liquid nitrogen. The pulverized powder was uniformly (with high agitation) dissolved in 1 mL TRIzol Reagent (Invitrogen, CA, USA). Chloroform (200 μL) was added, and the solution further shaken thoroughly. It was then kept on ice for 5 min. Centrifugation (10,000×g, 15 min) was carried out before 1:1 (v/v) of supernatant, and isopropyl alcohol was uniformly and gently mixed with a pipette. The mixture was returned to the ice for 20 min, then centrifuged (10,000×g, 30 min). The supernatant was carefully discarded, 1 ml of 70% ethanol pipetted into the tubes to wash the residual pellet by gently allowing the ethanol to flow over the pellets, while partially rotating the tubes. The ethanol was then discarded carefully. The wash-discard process repeated twice; then, the tubes were air-dried for 5 min. The RNA pellets were then dissolved in 80 μL of RNase-free water. The quality and quantity of the RNA were assessed by NanoDrop 2000/2000c (Thermo Scientific, Wilmington, DE, USA) and RNA integrity number (RIN) by Agilent 2100 Bioanalyzer (Agilent Technologies, Santa Clara, CA, USA). The mixture was then stored at − 80 °C [22]. Before RNA sequencing, 10 μg of total RNA from all samples was first treated RNase-free DNase I (Takara, Japan) to digest DNA remnants. RiboZero rRNA removal kit (Epicentre, USA) for gram-positive was used to eliminate ribosomal RNA before RNA sequencing analysis was done. One hundred nanograms of rRNA-less RNA from each test were fractionated into 200~300 nucleotides (nts) and utilized as a format for random prepared PCR. Strand-specific cDNA libraries were generated by standard procedures for ensuing Illumina sequencing using the mRNA-seq Test Prep pack (Illumina, USA) system platform (Shanghai Oebiotech Co., Ltd., Shanghai, China).

### Transcriptome data analysis

Duplicated sequences, ambiguous reads, and low-quality reads (Q > 20) were removed and assembled from the raw reads. Then the clean reads were mapped against the reference genome *B. velezensis* FZB42 (GenBank accession code: NC_009725.1) using the Burrows-Wheeler transform (BWT) algorithm [57]. To analyse, differentially expressed genes (DEGs) levels with a different nitrogen source, the number of reads were calculated by using Reads Per Kilobase per Million Mapped Reads (RPKM) [58]. The Upper Quartile (UQ) normalization factors were applied to the samples and multiplied by the mean upper quartile by applying the FDR adjustment with the threshold of < 0.05 q-values to compare different experiments or samples using Rockhooper2 [59]. Differential expression analysis of two conditions/groups (two biological replicates per condition) was performed using the DESeq R package (1.18.0) [60]. To analyze functional annotation, all the unigenes were compared by the evolutionary genealogy of genes: Clusters of Orthologous Groups (COG) and Kyoto Encyclopedia of Genes and Genomes (KEGG) under the cutoff of *P*-value < 0.05 using BLAST program [61, 62].

### Gene expression analysis by quantitative RT-PCR

Further to validate the transcriptomic data between the different nitrogen concentrations, 8 DEGs that including *glnK, glnL, mnrA, ywnA1, ydeB, narI, thrB*, and *thrC* were selected to analyse by quantitative real-time RT-PCR (RT-qPCR). Total RNA of each sample (the same sample used in RNA-seq) was first reverse-transcribed by random primer. Obtained cDNAs were further applied for RT-qPCR. The RT-qPCR reactions were carried out using the SYBR Premix Ex Taq kit (Takara, Japan) in a StepOne Real-Time PCR System (Applied Biosystems, Carlsbad, CA), according to the manufacturer’s instructions. Specific qPCR primers were designed by Primer-BLAST (https://www.ncbi.nlm.nih.gov/tools/primer-blast) based on the target sequences and synthesized by Shanghai Shenggong Biotechnology Co., Ltd., China (Additional File 11). The RT-qPCR reaction mixtures were prepared in triplicates using 10 μl of 2 × Power SYBR® Green PCR Master Mix (Applied Biosystems™) containing 1 μl of each primer (0.4 μM), 1 μl of cDNA and 7 μl ddH_2_O. PCR amplification was performed under the following conditions: 95 °C for 5 min, followed by 45 cycles of 95 °C for 10 s, 57 °C for 10 s and 72 °C for 10 s, finally at 95 °C for 15 s, for dissociation curve analysis, continued with 60 °C for 60 s and 95 °C for 10 s. The relative expression ratio of the target genes versus the 16S rDNA gene was calculated using the 2^-ΔΔCT^ method, and all data were given in terms of relative mRNA expression [63].

### Construction of *glnK* and *mnrA* knockout LG37 strains

The mutant Δ*glnK* strain with *glnK* deletion and Δ*mnrA* with *mnrA* deletion were constructed with chimeric single guide RNA (sgRNA) using the plasmid pJOE8999 and the homologous recombination methods as described [51]. The 20-nt protospacer adjacent motif (PAM) sequence was determined with 20 bp upstream of 5′-NGG-3′ in *glnK* and *mnrA* sequences of *B. velezensis* LG37, and the primers were designed and synthesized by Shanghai Sangon Biotechnology Co., Ltd. (Additional File 11). The pJOE8999 plasmid was digested with *Bsa*I restriction enzyme. Both products of primers sgRNA-*glnK* and sgRNA-*mnrA* were then ligated to generate pJOE8999-sgRNA-*glnK* and pJOE8999-*mnrA* plasmids, respectively.

The backbone of the vector and the spacer sequence was integrated by using 600 bp of upstream and 600 bp of downstream (UD) DNA fragments respective to *glnK* and *mnrA* amplified from the LG37 genomic DNA by homologous exchange fragments (HEFs). The complete *glnK* and *mnrA* HEFs were obtained by gene splicing by overlap extension PCR (SOEPCR). The obtained two complete HEFs sequences of *glnK* and *mnrA* were inserted into pJOE8999 plasmid in the *Sfi*I recognition site by *Sfi*I restriction endonuclease cleavage to accomplish the integrating plasmid pJOE8999-sgRNA-*glnK*UD and pJOE8999-sgRNA-*mnrA*UD. The fidelity of selected mutants of sgRNA DNA and HEF inserts were verified by sequencing and restriction enzyme digestion analysis. The pJOE8999 vector has a kanamycin resistance gene (*kanR*) encodes KanRP as a reporter protein and possesses a kanamycin resistance marker for convenient screening. The detailed procedures were illustrated in Additional File 8.

### Construction of *glnK* and *mnrA* overexpression LG37 strains

To investigate the exact role of *glnK* and *mnrA* in ammonia assimilation, the P_*xyl*_ promoter DNA region from LG37 genomic DNA was amplified, and the PCR products were inserted into the pHT1K expression vector on *Nco*I and *Bam*HI restriction endonuclease recognition site. Subsequently, PCR amplified products of LG37 genomic *glnK* and *mnrA* were cloned into the integrating plasmid pHT1K-P_*xyl*_ at *Bam*HI and *Kpn*I restriction endonuclease recognition site to obtain pHT1K-P_*xyl*_-*glnk* and pHT1K-P_*xyl*_-*mnrA* recombinant plasmids, respectively. The DNA sequences were amplified using primers listed in (Additional File 11). The acquired plasmids were transformed into competent *Escherichia coli* DH5*α* cells for overnight cultivation in LB ampicillin plates at 37 °C. Positive clones containing inserts of the expected size for P_*xyl*_, *glnK,* and *mnrA* fragments were verified by sequencing and by using specific restriction enzyme digestion analysis. Both recombinants were transformed separately into LG37 by electroporation, and successful transformants were screened with 25 μg/mL erythromycin in LB solid medium. Diagrammatic representation of a typical OE plasmid construction was shown in Additional File 9.

### Statistical analysis

Statistical analyses were performed using a statistical package for social sciences (SPSS, 18.0) software, and all data were represented as mean ± standard deviation (*SD*) of three independent experiments. The statistical significance was assessed by one-way analysis of variance (ANOVA), and the figures were drawn in Origin 9. Values *p* < 0.05 (*) was considered to be statistically significant difference and *p* < 0.01 (**) value as extremely difference, whereas *p* > 0.05 as not significant values.

## Supplementary information

**Additional file 1 Table S1.** Composition of Minimal media.

**Additional file 2 Table S2.** List of primers sequences used in this study.

**Additional file 3 Figure S1.** Construction of LG37 - ΔglnK and ΔmnrA mutant strains. (A) The physical map of CRISPR-Cas9 vector pJOE8999 (Altenbuchner, 2016) [52] (B) The targeted sgRNA sequences of glnK and mnrA containing 20 bp guide sequence with 5′NGG upstream along with the Bsa I (Bold) restrictions at both the ends. (C) The homologous exchange fragments of glnK (glnKUD) and mnrA (mnrAUD) consist of 600 bp upstream and downstream, respectively along with the SfiI restriction sites (Bold) at both the ends to link the spacer sequences.

**Additional file 4 Figure S2.** Physical map structure of pHT1K-P_xyl_ vector and construction of overexpression plasmids (pHT1K-P_xyl_-glnK and pHT1K-P_xyl_-mnrA). Schematic diagram showing the *E. coli* origin, an ampicillin resistance gene (AMP^R^), pHT1K under the control of P_xyl_ promoter (Pink), and the genes of interest (glnK and mnrA, Blue) in between the Kpn I and BamH I restriction endonuclease recognition site.

**Additional file 5 Figure S3.** Growth curves of LG37 generated to determine the optimal culture conditions. A: temperature (25, 28, 30, 32, 35, 37 °C), B: salinity (1, 2, 3, 4, 5, 6%), C: pH (5, 6, 7, 7.5, 8, 8.5, 9, 9.5), D: dissolved oxygen (DO; 1.8, 3, 4.2, 6.0 mg/L).

**Additional file 6 Table S3.** Summary statistics of sequencing library.

**Additional file 7 Table S4.** Summary of assembly and prediction of LG37.

**Additional file 8 Table S5.** The candidate related genes of NH_4_^+^ metabolism.

**Additional file 9 Table S6.** All the identified DEGs in this study by Gene Ontology terms.

**Additional file 10 Table S7.** All the identified DEGs in this study by Kyoto Encyclopedia of Genes and Genomes.

**Additional file 11 Table S8.** List of randomly selected DEGs for RT-qPCR.

## Data Availability

RNA-seq date have been submitted to GEO under the accession number GSE136178.

## Abbreviations

CRISPR-Cas: The clustered regularly interspaced short palindromic repeat (CRISPR)-Associated (Cas) systems
DEGs: Differentially expressed genes
GO: Gene ontology
KEGG: Kyoto Encyclopedia of Genes and Genomes
KOG: EuKaryotic
LG37: *Bacillus velezensis* LG37
OD: Optical density
RT-qPCR: Quantitative Polymerase Chain Reaction
