# Supplementary material for: Bacillus velezensis LG37: transcriptome profiling and functional verification of GlnK and MnrA in ammonia assimilation
Source: BMC Genomics. 2020 Mar 6;21:215. doi: 10.1186/s12864-020-6621-1 (PMC7060608; doi:10.1186/s12864-020-6621-1)
Supplement: Supplementary file 1 — Additional file 1 Table S1. Composition of Minimal media. [file 12864_2020_6621_MOESM1_ESM.docx]

**Table S1**

Summary statistics of sequencing library.

| **Sample** | **Raw_reads** | **Clean_reads** | **Valid_bases** | **GC** |
| --- | --- | --- | --- | --- |
| Gln-N1 | 19399436 | 19091060 | 93.61% | 47.27% |
| Gln-N2 | 19558394 | 19270408 | 93.91% | 47.40% |
| Gln-N3 | 19366106 | 19079520 | 93.18% | 47.04% |
| NH_4_^+^-N1 | 19436332 | 18922170 | 92.68% | 46.87% |
| NH_4_^+^-N2 | 19320848 | 18818298 | 92.68% | 46.85% |
| NH_4_^+^-N3 | 19399846 | 18865012 | 92.57% | 46.72% |
