# Supplementary material for: Bacillus velezensis LG37: transcriptome profiling and functional verification of GlnK and MnrA in ammonia assimilation
Source: BMC Genomics. 2020 Mar 6;21:215. doi: 10.1186/s12864-020-6621-1 (PMC7060608; doi:10.1186/s12864-020-6621-1)
Supplement: Supplementary file 2 — Additional file 2 Table S2. List of primers sequences used in this study. [file 12864_2020_6621_MOESM2_ESM.docx]

**Table S2**

Summary of assembly and prediction of LG37.

| **Statistic item** | **Number** |
| --- | --- |
| Number of predicted transcripts (not antisense) | 30 |
| Number of new predicted transcripts | 63 |
| Number of predicted multi-gene operons | 759 |
| Number of predicted RNAs (antisense) | 33 |
| Number of differentially expressed protein coding genes | 2569 |
| Number of 5’UTR | 2131 |
| Number of 3’UTR | 2037 |
