# Supplementary figures and images for: Bacillus velezensis LG37: transcriptome profiling and functional verification of GlnK and MnrA in ammonia assimilation
Source: BMC Genomics. 2020 Mar 6;21:215. doi: 10.1186/s12864-020-6621-1 (PMC7060608; doi:10.1186/s12864-020-6621-1)

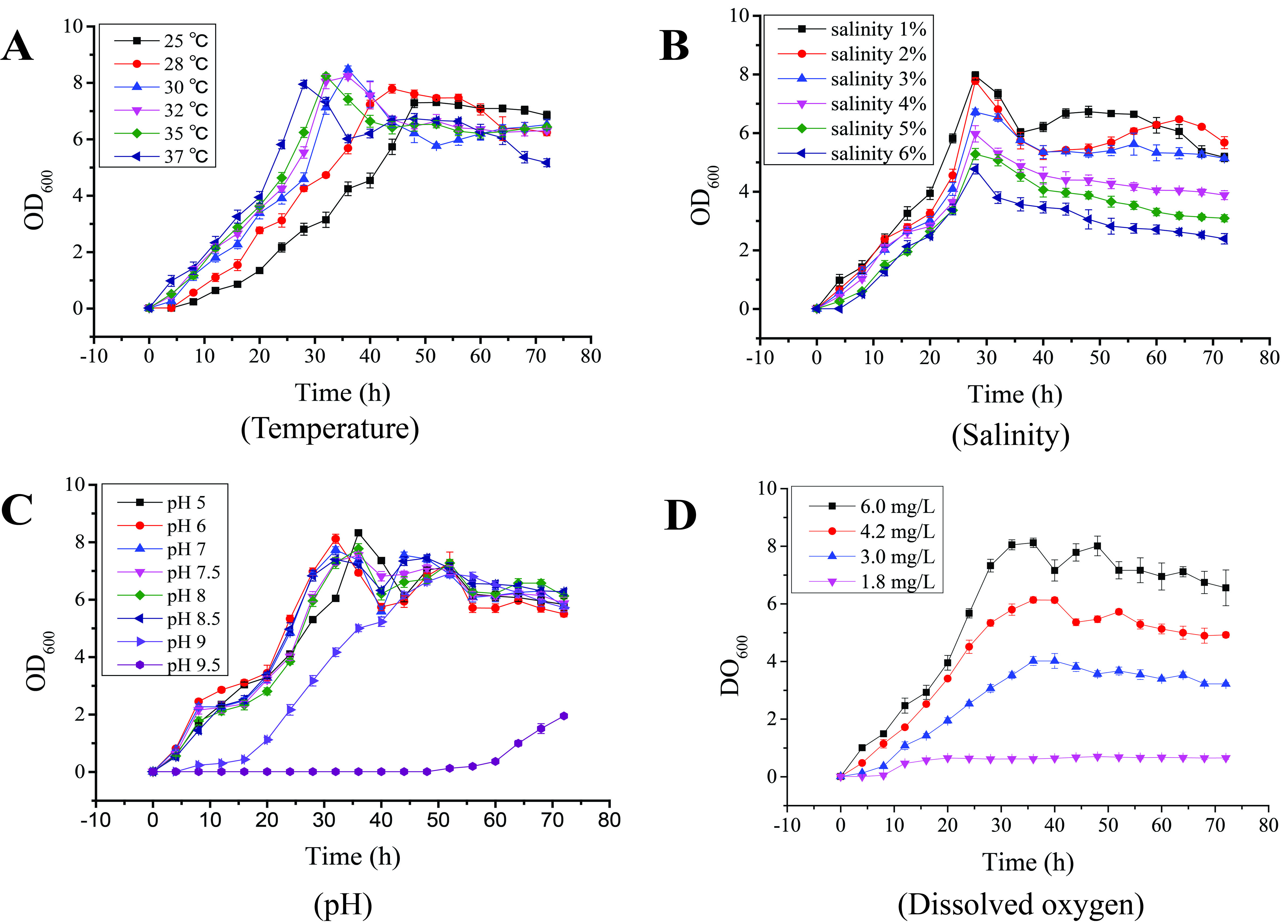

Supplement: Supplementary file 3 — Additional file 3 Figure S1. Construction of LG37 - ΔglnK and ΔmnrA mutant strains. (A) The physical map of CRISPR-Cas9 vector pJOE8999 (Altenbuchner, 2016) [52] (B) The targeted sgRNA sequences of glnK and mnrA containing 20 bp guide sequence with 5′NGG upstream along with the Bsa I (Bold) restrictions at both the ends. (C) The homologous exchange fragments of glnK (glnKUD) and mnrA (mnrAUD) consist of 600 bp upstream and downstream, respectively along with the SfiI restriction sites (Bold) at both the ends to link the spacer sequences. [file 12864_2020_6621_MOESM3_ESM.jpg]

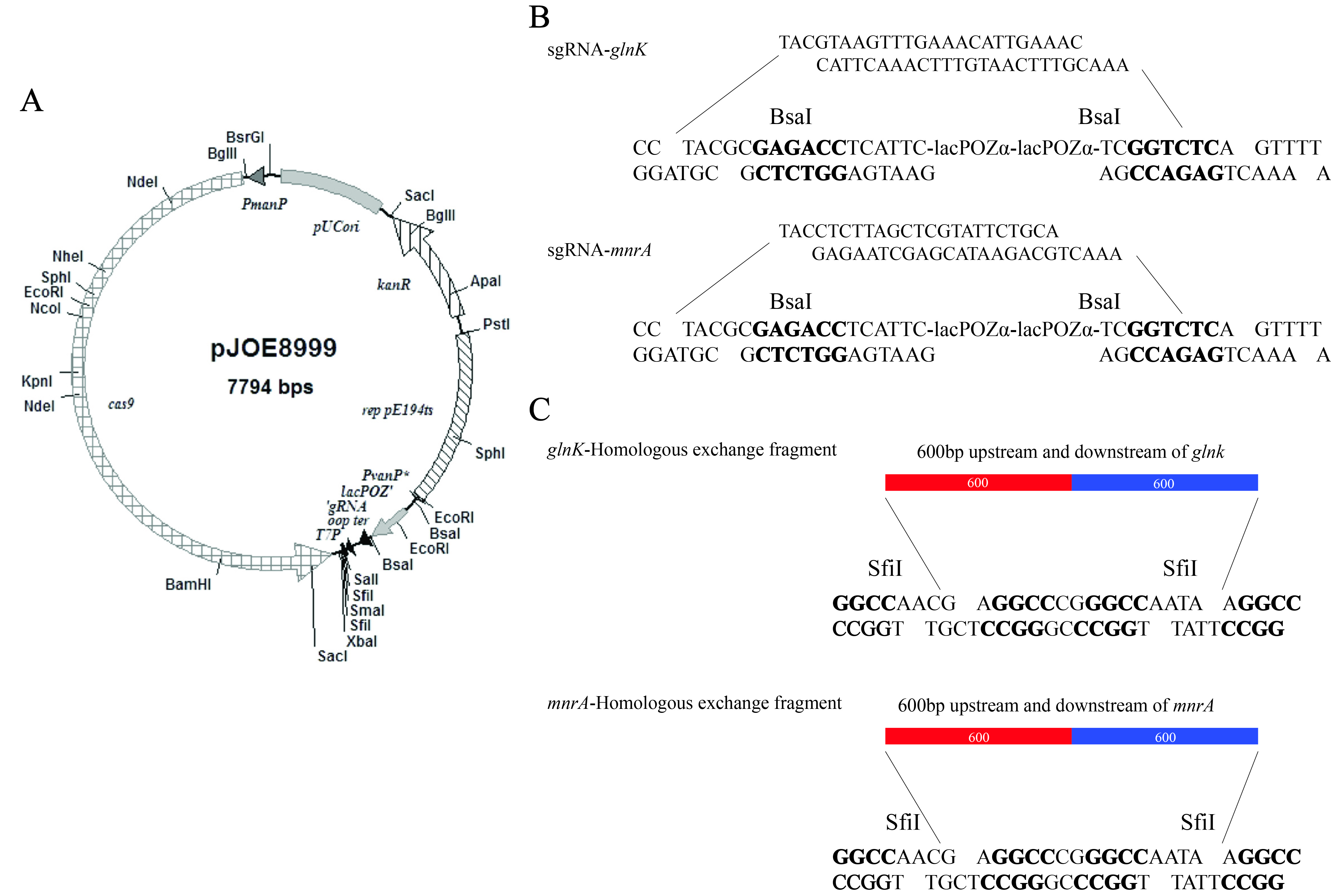

Supplement: Supplementary file 4 — Additional file 4 Figure S2. Physical map structure of pHT1K-Pxyl vector and construction of overexpression plasmids (pHT1K-Pxyl-glnK and pHT1K-Pxyl-mnrA). Schematic diagram showing the E. coli origin, an ampicillin resistance gene (AMPR), pHT1K under the control of Pxyl promoter (Pink), and the genes of interest (glnK and mnrA, Blue) in between the Kpn I and BamH I restriction endonuclease recognition site. [file 12864_2020_6621_MOESM4_ESM.jpg]

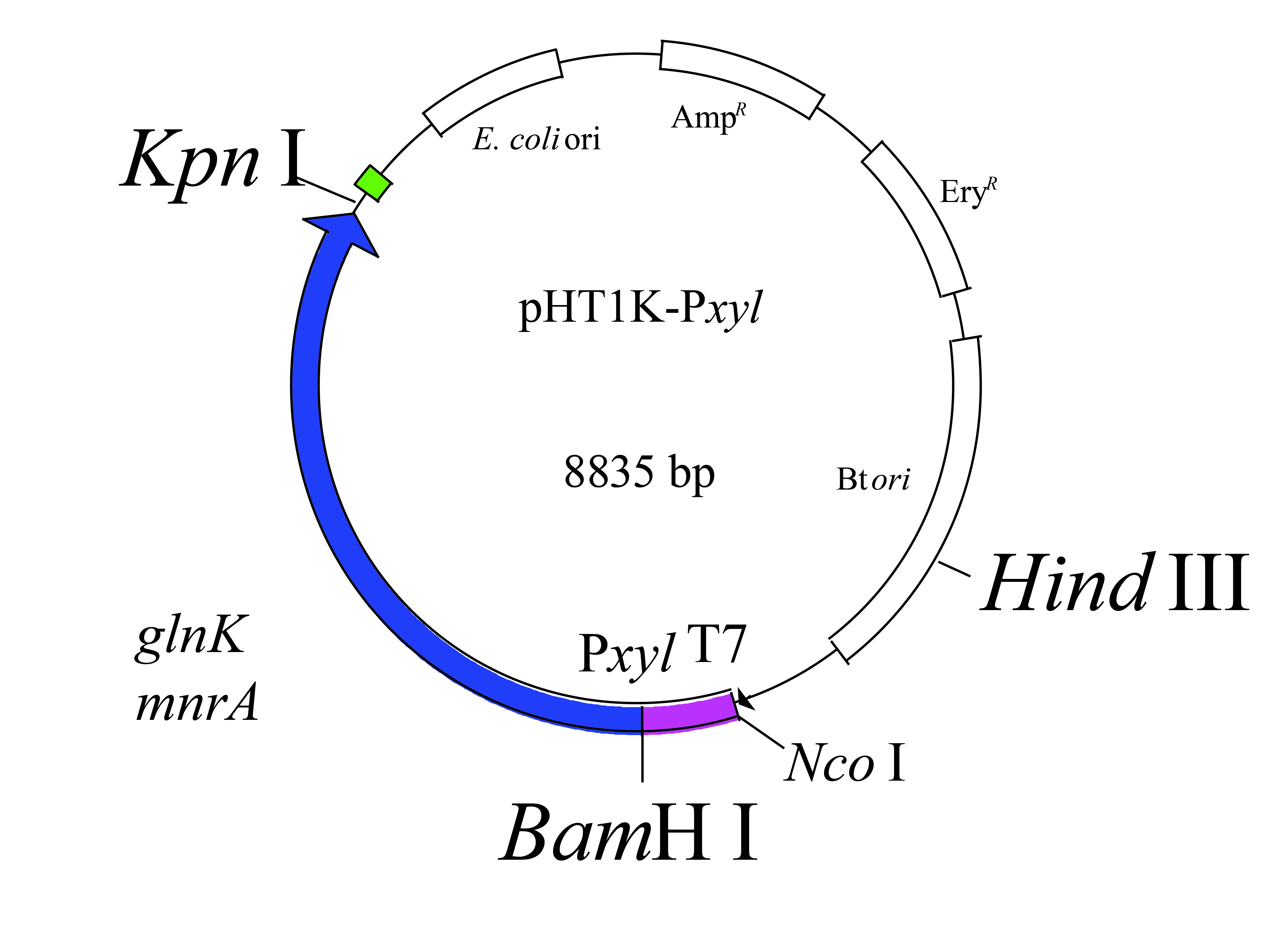

Supplement: Supplementary file 5 — Additional file 5 Figure S3. Growth curves of LG37 generated to determine the optimal culture conditions. A: temperature (25, 28, 30, 32, 35, 37 °C), B: salinity (1, 2, 3, 4, 5, 6%), C: pH (5, 6, 7, 7.5, 8, 8.5, 9, 9.5), D: dissolved oxygen (DO; 1.8, 3, 4.2, 6.0 mg/L). [file 12864_2020_6621_MOESM5_ESM.jpg]
