# Supplementary material for: Bacillus velezensis LG37: transcriptome profiling and functional verification of GlnK and MnrA in ammonia assimilation
Source: BMC Genomics. 2020 Mar 6;21:215. doi: 10.1186/s12864-020-6621-1 (PMC7060608; doi:10.1186/s12864-020-6621-1)
Supplement: Supplementary file 6 — Additional file 6 Table S3. Summary statistics of sequencing library. [file 12864_2020_6621_MOESM6_ESM.docx]

**Additional File 4**

The candidate related genes of NH_4_^+^ metabolism.

| **Gene ID** | **Product** | **Fold change (*q-*value<0.05)** | |
| --- | --- | --- | --- |
|  |  | **Up-regulated** | **Down-regulated** |
| *orf03938* | Sensor histidine kinase GlnK | 4.26 |  |
| *orf03939* | DNA-binding response regulator GlnL | 4.12 |  |
| *orf03940* | Hypothetical protein | 3.83 |  |
|  | DUF2651 domain-containing protein | 3.50 |  |
| *orf03625* | Hypothetical protein | 3.13 |  |
| *orf03916* | Hypothetical protein | 3.08 |  |
| *orf02113* | Hypothetical protein | 3.08 |  |
| *orf03634* | MFS transporter MnrA | 2.93 |  |
| *orf03936* | ABC transporter permease NatB | 2.76 |  |
|  | Hypothetical protein | 2.46 |  |
| *orf03631* | Rrf2 family transcriptional regulator YwnA | 2.39 |  |
| *orf00656* | Hypothetical protein | 2.33 |  |
| *orf03630* | NAD(P)-dependent oxidoreductase YwnB | 2.28 |  |
| *orf00188* | Hypothetical protein | 2.22 |  |
| *orf03633* | Hypothetical protein | 2.20 |  |
| *orf03626* | CarD family transcriptional regulator YdeB | 2.19 |  |
| *orf03937* | ABC transporter ATP-binding protein NatA | 2.04 |  |
| *orf00488* | DNA-binding response regulator | 2.00 |  |
| *orf00068* | CtsR family transcriptional regulator CtsR |  | -2.04 |
| *orf01620* | SAM-dependent methyltransferase RamA |  | -2.07 |
| *orf00581* | Serine hydroxymethyltransferase GlyA |  | -2.08 |
| *orf01102* | Threonine synthase ThrC |  | -2.20 |
| *orf01103* | Homoserine kinase ThrB |  | -2.32 |
| *orf01101* | Homoserine dehydrogenase HoM |  | -2.86 |
| *orf00547* | Nitrate reductase subunit gamma NarI |  | -2.89 |
| *orf01234* | DinB family protein DinB |  | -2.99 |
| *orf01440* | Murein hydrolase regulator LrgA |  | -5.61 |
