# Supplementary material for: Bacillus velezensis LG37: transcriptome profiling and functional verification of GlnK and MnrA in ammonia assimilation
Source: BMC Genomics. 2020 Mar 6;21:215. doi: 10.1186/s12864-020-6621-1 (PMC7060608; doi:10.1186/s12864-020-6621-1)
Supplement: Supplementary file 9 — Additional file 9 Table S6. All the identified DEGs in this study by Gene Ontology terms. [file 12864_2020_6621_MOESM9_ESM.docx]

**Additional File 7**

The DEGs for RT-qPCR.

| **Gene ID** | **Product** | **Fold change (*q*-value < 0.05)** | |
| --- | --- | --- | --- |
|  |  | **Up-regulated** | **Down-regulated** |
| *orf03938* | Sensor histidine kinase - GlnK | 4.26 |  |
| *orf03939* | DNA-binding response regulator - GlnL | 4.12 |  |
| *orf03634* | MFS transporter - MnrA | 2.92 |  |
| *orf03631* | Rrf2 family transcriptional regulator - YwnA | 2.39 |  |
| *orf03626* | CarD family transcriptional regulator - YdeB | 2.19 |  |
| *orf00547* | Nitrate reductase subunit gamma - NarI |  | -2.89 |
| *orf01102* | Threonine synthase - **ThrC** |  | -2.20 |
| *orf01103* | Homoserine kinase - **ThrB** |  | -2.32 |
