# Supplementary material for: Bacillus velezensis LG37: transcriptome profiling and functional verification of GlnK and MnrA in ammonia assimilation
Source: BMC Genomics. 2020 Mar 6;21:215. doi: 10.1186/s12864-020-6621-1 (PMC7060608; doi:10.1186/s12864-020-6621-1)
Supplement: Supplementary file 10 — Additional file 10 Table S7. All the identified DEGs in this study by Kyoto Encyclopedia of Genes and Genomes. [file 12864_2020_6621_MOESM10_ESM.docx]

**Table S7**

Composition of Minimal medium.

| **Components Conc. (g/L)** | | **1000 × trace elements**  **Components Conc. (g/L)** | |
| --- | --- | --- | --- |
| Glucose | 18.0 | CaCl_2_•2H_2_O | 0.73 |
| KH_2_PO_4_ | 13.1 | ZnCl_2_ | 0.17 |
| K_2_HPO_4_ | 1.2 | CuCl_2_•2H_2_O | 0.043 |
| Na3C6H5O7•2H2O | 0.05 | CoCl_2_•6H_2_O | 0.006 |
| MgSO•4H_2_O | 1.0 | Na_2_MoO_4_•2H_2_O | 0.006 |
| 1000 × trace elements | 1 ml | FeCl_2_•4H_2_O | 1.35 |
| NH_4_^+^ / glutamine-N | N mmol/L | MnCl_2_•4H_2_O | 0.01 |

**Note:** ‘N’ denotes the different concentrations of NH_4_^+^/glutamine-N.
