# Supplementary material for: Bacillus velezensis LG37: transcriptome profiling and functional verification of GlnK and MnrA in ammonia assimilation
Source: BMC Genomics. 2020 Mar 6;21:215. doi: 10.1186/s12864-020-6621-1 (PMC7060608; doi:10.1186/s12864-020-6621-1)
Supplement: Supplementary file 11 — Additional file 11 Table S8. List of randomly selected DEGs for RT-qPCR. [file 12864_2020_6621_MOESM11_ESM.docx]

**Additional File 11**

List of primers sequences used in this study.

| **Gene** | **Primer sequence** |
| --- | --- |
| sgRNA-*glnK* | TACG TTCAGGACTATACTACTCAA  AAGTCCTGATATGATGAGTT CAAA |
| sgRNA-*mnrA* | TACG TTCCTACCTATGATCAGATT  AAGGATGGATACTAGTCTAA CAAA |
| **PCR assays** |  |
| *glnK*-F | ATGAAATTAATTGATTATGTGAAA |
| *glnK*-R | CTAATTACTTATTGCTTCATTTGC |
| *glnK* (Upstream)-F | GAATTTTGAAACTGTTATATGCGG |
| *glnK* (Upstream)-R | TCATTAGATTATCCCCTGTTTTTAA-TGAATTATATATCCCATTCCTTTT |
| *glnK* (Downstream)-F | AAAGGAATGGGATATATAATTCA-TTAAAAACAGGGGATAATCTAATG |
| *glnK* (Downstream)-R | TATTACTTTTCTCCAAAAAGATAA |
| *glnK* (sfil)--F | TTGGCCAACGAGGCCATGAAATTAATTGATTATGTGAAA |
| *glnK* (sfil)--R | TTGGCCTTATTGGCCTATTACTTTTCTCCAAAAAGATAA |
| *mnrA*-F | ATGAAGTTTAACAAGAAAAAAATT |
| *mnrA* -R | TCATTCCTTTTCTGTTTGTTCCAT |
| *mnrA* (Upstream)-F: | ATGGTACGTTTATAGTGTTTTGTT |
| *mnrA* (Upstream)-R | CCGGTCAATTAAATGGATGTTTTTT-TTAATAATATCGACTCCTTAATTA |
| *mnrA* (Downstream)-F | ATAATTAAGGAGTCGATATTATTAA-AAAAAACATCCATTTAATTGACCG |
| *mnrA* (Downstream)-R | TTTAATTTCTTCTTCATTACAACC |
| *mnrA* (sfil)-F | TTGGCCAACGAGGCCATGGTACGTTTATAGTGTTTTGTT |
| *nmtA* (sfil)-R | TTGGCCTTATTGGCCTTTAATTTCTTCTTCATTACAACC |
| P*xyl* (*Nco* I)-F: | CATGCCATGGATTCCTCGTGAAGTAAAGTATGTAT |
| P*xyl* (*Bam*HI)-R: | CGCGGATCCGTGATTTCCCCCTTAAAAATAAATT |
| **RT-qPCR assays** |  |
| *16S rRNA*-F | GTGAGGTAACGGCTCACCAA |
| *16S rRNA*-R | AAGGTGCCGCCCTATTTGAA |
| *glnK*-F | GGGGGAACGCTGACTATTACA |
| *glnK*-R | ACCCTCTACGCTGTCTACCT |
| *glnL*-F | AGCCATGTATTGGTGGCGAA |
| *glnL*-R | CCGCGAAACAACTTTCCCTT |
| *mnrA* -F | TTTGCTTAGACCGGTAGGCG |
| *mnrA* -R | ATTGCACCAGCGTACTCTCC |
| *ywnA*-F | AATGTTCGGCCAGGTATTGC |
| *ywnA*-R | CCGGCCTACAGGACATTGAG |
| *ydeB*-F | AATGCACGGAGCAGGTACAA |
| *ydeB*-R | TGCTTCCATGGCAGTAACCT |
| *narI*-F | CGGACAGATCCTCTGGGGTA |
| *narI*-R | ACGCCTTCCGGGATCAAAAT |
| *thrC*-F | AGCTGATTGCCCGTGAAGAA |
| *thrC*-R | ACAGCCGTGTTCGGATCTTT |
| *thrB*-F | CATCCTCCATCAGCCGTACC |
| *thrB*-R | TCACAGTGGGGGAAATGCTC |
| *glnA*-F | GCCGGTATCGTAAAACACGC |
| *glnA*-R | GCAATACGCTTAACGCCAGG |
| *amtB*-F | GGGCTGGCTTTGTTTTACGG |
| *amtB*-R | AACAAGGAATGCGGGATCGT |
| *glnB*-F | TTACGCGTCCGGCTAAGTTT |
| *glnB*-R | TTTTTCGCCGTTTCCACGAC |
|  |  |
